# Loss of function of the ALS protein SigR1 leads to ER pathology associated with defective autophagy and lipid raft disturbances

**DOI:** 10.1038/cddis.2014.243

**Published:** 2014-06-12

**Authors:** J T Vollrath, A Sechi, A Dreser, I Katona, D Wiemuth, J Vervoorts, M Dohmen, A Chandrasekar, J Prause, E Brauers, C M Jesse, J Weis, A Goswami

**Affiliations:** 1 Institute of Neuropathology, Uniklinik RWTH Aachen and JARA Brain Translational Medicine, Pauwelsstraße 30, Aachen 52074, Germany; 2 Institute of Biomedical Engineering and Cell Biology, RWTH Aachen University and JARA Brain Translational Medicine, Pauwelsstraße 30, Aachen 52074, Germany; 3 Institute of Physiology, RWTH Aachen University, Pauwelsstraße 30, Aachen 52074, Germany; 4 Institute of Biochemistry and Molecular Biology, RWTH Aachen University, Pauwelsstraße 30, Aachen 52074, Germany

## Abstract

Intracellular accumulations of altered, misfolded proteins in neuronal and other cells are pathological hallmarks shared by many neurodegenerative diseases including amyotrophic lateral sclerosis (ALS). Mutations in several genes give rise to familial forms of ALS. Mutations in Sigma receptor 1 have been found to cause a juvenile form of ALS and frontotemporal lobar degeneration (FTLD). We recently described altered localization, abnormal modification and loss of function of SigR1 in sporadic ALS. In order to further elucidate the molecular mechanisms underlying SigR1-mediated alterations in sporadic and familial ALS, we extended our previous studies using neuronal SigR1 knockdown cell lines. We found that loss of SigR1 leads to abnormal ER morphology, mitochondrial abnormalities and impaired autophagic degradation. Consistent with these results, we found that endosomal trafficking of EGFR is impaired upon SigR1 knockdown. Furthermore, in SigR1-deficient cells the transport of vesicular stomatitis virus glycoprotein is inhibited, leading to the accumulation of this cargo protein in the Golgi apparatus. Moreover, depletion of SigR1 destabilized lipid rafts and associated calcium mobilization, confirming the crucial role of SigR1 in lipid raft and intracellular calcium homeostasis. Taken together, our results support the notion that loss of SigR1 function contributes to ALS pathology by causing abnormal ER morphology, lipid raft destabilization and defective endolysosomal pathways.

ER stress causes alterations in protein quality control, autophagy, calcium imbalance and mitochondrial dysfunction.^1, 2, 3^ It is central to the pathogenesis of many neurodegenerative diseases.^4, 5^ Altered proteins are targeted by molecular chaperones for protein repair or refolding. However, failure of this first line of defense leads to abnormal aggregates of such proteins that then form ubiquitinated cellular inclusions and compromise UPS function.^6, 7, 8, 9^ Macroautophagy, the major lysosomal degradative pathway in cells, is responsible for degrading long-lived cytoplasmic constituents; it is the principal mechanism for turning over cellular organelles and protein aggregates too large to be degraded by the proteasome.^10, 11, 12, 13^ Macroautophagy, hereafter referred to as autophagy, is a multistep process, initiated primarily by sequestration of portions of the cytoplasm in double-membrane-bound vesicles to form an autophagosome. Autophagosomes along with their cargoes are then degraded upon fusing with late endosome- or lysosome-containing cathepsins, other acid hydrolases, and vacuolar [H+] ATPase.^14^ Even though autophagy is a selective and efficient mechanism for the degradation of misfolded and mutant proteins related to neurodegeneration, recent evidence indicates that the alterations in certain disease-related genes may actually impair autophagic activity at different levels, including accumulation of autophagic vacuoles,^15^ substrate recognition, lysosomal acidity and autophagosome membrane nucleation.^16, 17^

SigR1 interacts with a variety of ligands and is involved in a broad array of biological functions that have only been partially defined so far. They include regulation of neuronal survival, neuritogenesis, ion channel activity, IP3R-mediated Ca^2+^ signaling, memory and drug addiction. SigR1 is an ER chaperone that is located at the mitochondria–ER interface and is normally bound to another chaperone, BiP/GRP78.^18^ Upon IP_3_ receptor stimulation or Ca^2+^ depletion within the ER, SigR1 dissociates from BiP and stabilizes IP_3_ receptors, leading to prolonged Ca^2+^ signaling into mitochondria.^18^ Recently, a mutation in SigR1, E1O2Q, has been reported to cause a juvenile form of amyotrophic lateral sclerosis (ALS) and frontotemporal lobar degeneration (FTLD).^19^ SigR1 protein is decreased significantly in human sporadic ALS spinal cord.^20^ In addition, functional relevance of SigR1 in ALS pathogenesis is demonstrated by SigR1 knockout mice that display motor deficits with a shorter latency period in rotarod experiments, and by another study showing that lack of SigR1 exacerbates ALS progression in SOD1 tg mice.^21, 22^ Furthermore, a recent study showed improvement in motor function and survival of motor neurons in SOD1 mice treated with a SigR1 agonist.^23^ Recently, we reported altered localization and abnormal modification of SigR1 in sporadic ALS and showed that loss of SigR1 function leads to deformities in ER structure, formation of ER-derived autophagic vacuoles and induction of ER stress.^20^ Loss of function of SigR1 also leads to aberrant calcium homeostasis and cell death.^20^ Together, these data suggest a crucial role of SigR1 in neuronal function and survival.

Autophagy has been known to be tightly linked to ER function and is decisive in neurodegeneration mediated by ER stress.^24, 25, 26^ Therefore, we hypothesized that loss of SigR1 function contributes to a vicious circle including ER stress, defective autophagy and altered calcium signaling that causes multifactorial ALS pathology. In the present study, we show that depletion of SigR1 leads to the accumulation of numerous autophagic vacuoles often filled with nondegraded autophagic substrates, and severe deformities in ER ultrastructure including loss of ER tethering. Biochemical analysis in SigR1-deficient cells revealed the accumulation of various autophagic substrates and defects in endosomal trafficking, suggesting an impairment of endolysosomal pathways. Finally, loss of SigR1 destabilized lipid rafts causing both impaired calcium mobilization and altered endosomal trafficking. Altogether, our results support the notion that loss of SigR1 contributes to ALS pathogenesis by causing abnormal ER morphology, lipid raft destabilization and defective autophagy.

## Results

### Loss of function of SigR1 leads to structural abnormalities of the ER/Golgi and to accumulation of autophagic material

**Figure 1:**
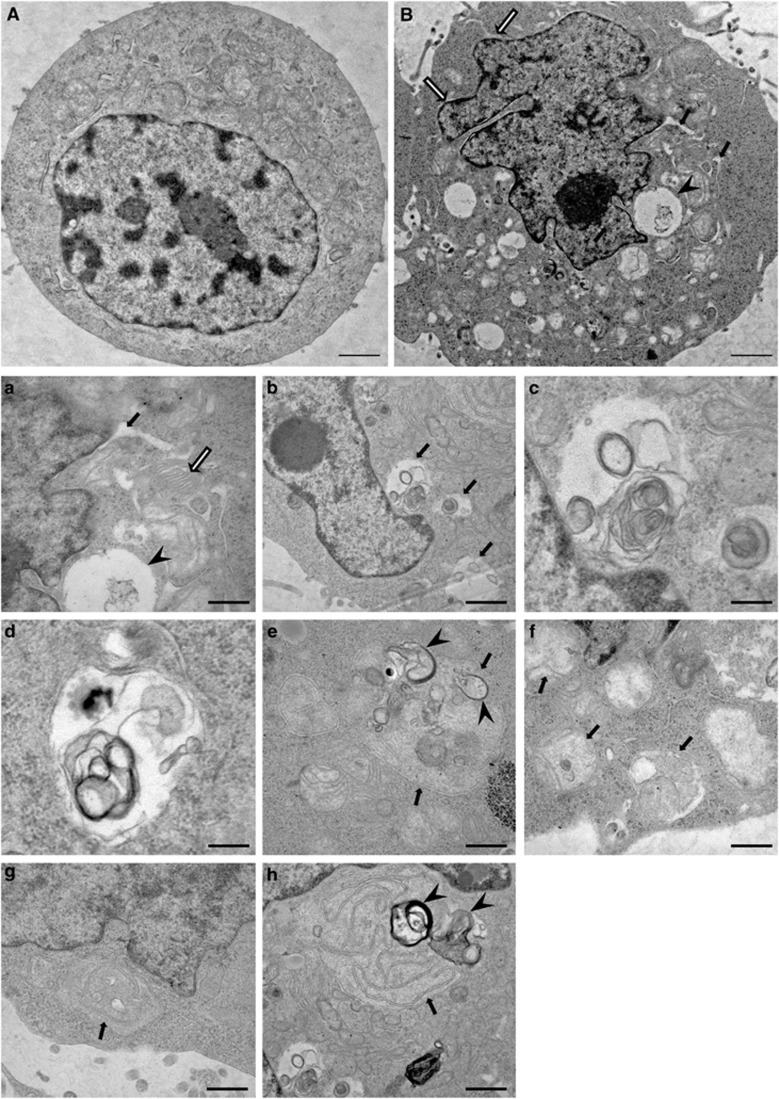
Depletion of SigR1 leads to ER deformities and accumulation of autophagic vacuoles. HEK293 cells were either transfected with control shRNA or SigR1 shRNA. After 48 h, the transfected cells were fixed with 2.5% buffered glutaraldehyde and processed for electron microscopy. (**A**) HEK293 cell transfected with control shRNA shows normal mitochondria and ER and no evidence of defective autophagy. Scale bar=1 *μ*m. (**B**) HEK293 cell transfected with SigR1 shRNA shows widened ER profiles (black arrows) and membrane-bound vacuolar structures, some corresponding to autophagosomes (arrowhead). Note the focal widening of the nuclear envelope (white arrows). Scale bar=1 *μ*m. (a) Higher magnification of the widened ER depicted in (**B**), which is now seen to be connected to the nuclear envelope (arrow), and stacked membranes (white arrow) probably derived from the Golgi. Arrowhead indicates incipient autophagosome. Scale bar=0.5 *μ*m. (b) Multiple lamellar bodies in large autophagic vacuoles (arrows) of another HEK293 cell transfected with SigR1 shRNA. Scale bar=1 *μ*m. (c) Enlarged view of one of the autophagic vacuoles depicted in (b), probably a mature double membranous autophagosome containing phospholipids and other undigested substrates. Scale bar=0.3 *μ*m. (d) Large, double membrane-bound autophagic vacuole containing membranous and other osmiophilic accumulations. Scale bar=0.3 *μ*m. (e) Enlarged mitochondrion (arrows) containing autophagic material indicating mitophagy. Scale bar=0.4 *μ*m. (f) Mitochondria (arrows) showing dissolution of cristae architecture. Scale bar=0.5 *μ*m. (g and h) Untethered ER (arrows) with several multilamellar bodies (arrowheads, only in h). Scale bar=0.5 *μ*m

We previously reported that knockdown of SigR1 in HEK 293 and NSC34 cells caused ER widening and induced the formation of numerous ER-derived vacuoles often filled with membranous autophagic material.^20^ In the present study, we confirmed our previous findings and further characterized these vacuolar structures in HEK 293 and NSC34 motor neuron-like cells lacking SigR1. EM studies revealed that depletion of SigR1 leads to the accumulation of numerous autophagic vacuoles^15^ often filled with nondegraded autophagic substrates (Figure 1B and Supplementary Figures 2A–D) and deformities of ER ultrastructure (Figure 1B). Furthermore, knockdown of SigR1 induced mitochondrial abnormalities as well as mitophagy and led to ER untethering and proliferation, suggesting a crucial role of SigR1 in maintaining structural integrity of the ER and stabilizing mitochondria at the MAM (Figure 1Be–h).

### Knockdown of SigR1 leads to the accumulation of various components of the lysosomal pathway

**Figure 2:**
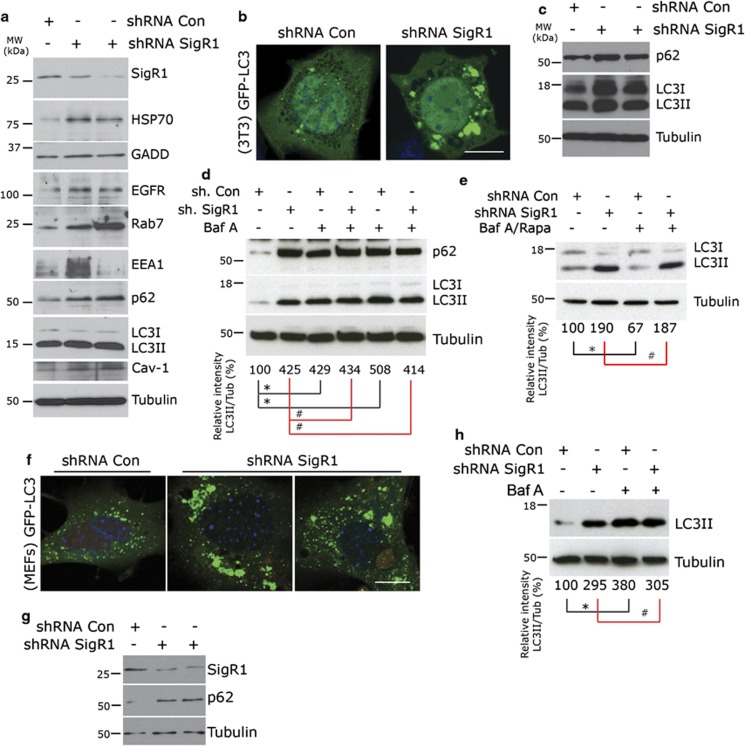
Accumulation of various components of the lysosomal pathway in SigR1-depleted cells. (**a**) NSC34 cells were transiently transfected with two different SigR1 shRNA constructs, achieving different levels of suppression of SigR1 expression. Cell lysates were subjected to immunoblot analysis with antibodies indicated. (**b**) NIH-3T3 cells stably expressing GFP-LC3 were transiently transfected either with control or SigR1 shRNA. At 48 h after transfection, cells were processed for immunofluorescence. Representative images depict the accumulation of GFP-LC3 as a marker for autophagy inhibition. Scale bar=8 *μ*m. (**c**) NIH-3T3 cells stably expressing GFP-LC3 were transiently transfected with two different SigR1 shRNA constructs, achieving different levels of suppression of SigR1 expression. Immunoblot analysis was performed using p62 and LC3 as markers for autophagy. (**d**) NIH-3T3 cells stably expressing GFP-LC3 were transiently transfected either with control or SigR1 shRNA. At 48 h after transfection, cells were treated with or without the autophagy inhibitor bafilomycin A at increasing concentration (lanes 3 and 4: 10 nM, lanes 5 and 6: 20 nM) for 3 h. Immunoblot analysis was performed using p62 and LC3 as markers for autophagy. (**e**) NIH-3T3 cells stably expressing GFP-LC3 were transiently transfected as described above. After 48 h, the cells were treated with or without the autophagy inhibitor bafilomycin A (20 nM) for 3 h, and then replaced by the autophagy inducer rapamycin (20 nM) for additional 3 h. Cell lysates were prepared and subjected to immunoblot analysis using the LC3 antibody. (**f**) MEFs obtained from GFP-LC3 tg mice were transiently transfected either with control or SigR1 shRNA. At 48 h after transfection, cells were processed for GFP staining. Scale bar=6 *μ*m. (**g**) Western blot analysis of these MEFs transiently transfected either with control or two different SigR1 shRNAs. At 48 h after transfection, cell lysates were subjected to p62 and LC3 immunoblots. (**h**) MEFs obtained from GFP-LC3 tg mice were transiently transfected either with control or SigR1 shRNA. At 48 h after transfection, cells were treated with or without the autophagy inhibitor bafilomycin A (20 nM) for 3 h. Cell lysates were prepared and subjected to immunoblot analysis using the LC3 antibody as a marker for autophagy. For all the experiments, values are means±S.D. of three independent experiments. *P<0.05, **P<0.005, ^#^not significant

Based on our observation that SigR1 deficiency induces the formation of defective ER and of ER-derived autophagic vacuoles, we hypothesized that the autophagic process is impaired in SigR1-deficient cells. Autophagy is characterized by the engulfment of cytoplasmic components^26^ in double-membrane-bound structures^25^ that are then delivered to lysosomes/vacuoles for degradation. Autophagic activity can thus be analyzed by determining the levels of LC3II and p62 by western blotting as well as the number of LC3II-positive cytoplasmic punctae by immunofluorescence.^10, 27, 28^ NSC-34 cells lacking SigR1 showed evidence of increased ER and misfolded protein stress (Figure 2a), confirming and extending our previous results,^20^ along with the accumulation of p62, LC3II and several other known markers for endolysosomal pathways such as epidermal growth factor receptor (EGFR), the early endosomal antigen EEA1 and Rab7 (Figure 2a and Supplementary Figure 1a). In order to further confirm that autophagy is defective, we generated a mouse fibroblast (NIH-3T3) cell line stably expressing GFP-LC3 with normal basal autophagic activity (Supplementary Figure 1d). Under basal conditions, knockdown of SigR1 in these cells led to abnormal accumulation of GFP-LC3 (Figure 2b). Abnormal autophagy was also evident by p62 and LC3 accumulation already in the absence of autophagy inhibitors (Figure 2c). Autophagy inhibition due to loss of SigR1 was further confirmed by the disproportionate increase in LC3II and of p62 levels in the presence of the known autophagy inhibitor bafilomycin A and of the autophagy inducer rapamycin (Figures 2d and e). Parallel experiments confirmed these results in HeLa cells transfected with a GFP-LC3 construct and obtained analogous results (Supplementary Figures 1b and c). In order to further confirm our results at a physiological expression level of LC3 in a primary cell culture system, we used MEFs from GFP-LC3 tg mice.^29^ Knockdown of SigR1 in MEFs confirmed the above-described results of disproportionate GFP-LC3 and p62 accumulation (Figures 2f–h), again consistent with the inhibition of autophagic degradation after SigR1 knockdown. Taken together, these results demonstrate that loss of SigR1 protein leads to a defective endolysosomal autophagy pathway.

### Depletion of SigR1 leads to defective endosome-mediated EGFR degradation/fusion of autophagosome to lysosomes

**Figure 3:**
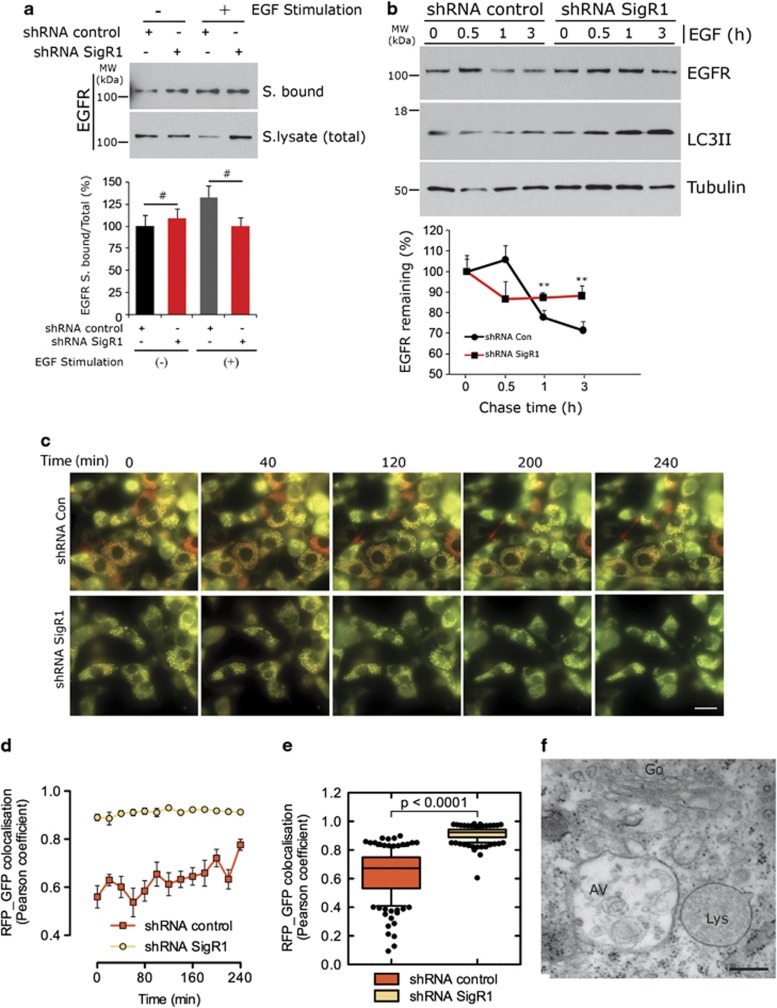
Defective endosome-mediated EGFR degradation/fusion of autophagosomes to lysosomes in SigR1 shRNA knockdown cells. (**a**) A431 cells were transiently transfected with either control shRNA or SigR1 shRNA. At 48 h after transfection, cells were processed for surface biotinylation assays. Immunoblot analysis using EGFR antibody revealed no significant alteration in EGFR internalization. Values in the quantification (lower panel) are means±S.D. of three independent experiments. ^#^not significant. (**b**) A431 cells were transiently transfected as described above and proceeded for EGFR degradation assays combined with LC3 immunoblots. Lower panel: densitometric analysis of EGFR immunoblots (Adobe PS CS5). Values are expressed as mean±S.D. from three independent experiments. **P<0.005. (**c**) NIH-3T3 cells stably expressing RFP-GFP-LC3 were transiently transfected either with control or SigR1 shRNA (without RFP tag, see Materials and Methods). At 48 h after transfection, cells were treated with bafilomycin A (20 nM) for 3 h to block autophagosome fusion; 1 h later, the rate of lysosome fusion with autophagosomes was monitored in control and SigR1 shRNA-transfected cells by live cell imaging. Scale bar=25 *μ*m. (**d** and **e**) The rate of autophagosome maturation reflected by Pearson's coefficient (green/red fluorescence ratio) at each time point indicated after 1 h of bafilomycin A removal. Values are represented as means±S.E.M. of triplicate experiments. (**f**) Large, double membrane-bound autophagic vacuole (AV) containing membranous and other osmiophilic accumulations, not fused with lysosomes (Lys) in SigR1 shRNA-transfected cells. Scale bar=0.25 *μ*m

Because the impaired degradation and accumulation of autophagic substrates was indicative of a deficient autophagic process, we hypothesized that the transport and fusion of endosomes/autophagosomes to lysosomes was impaired. We took advantage of the widely studied internalization and degradation of the EGF–EGFR complex in A431 cells expressing high levels of EGFR and showing normal autophagic activity (Supplementary Figure 1e). In this system, EGFR can be activated by both EGF and TGF-*α*,^30, 31^ leading to clustering and internalization of the EGF–EGFR complex into early endosomes or membrane compartments characterized by tubular vesicular morphology and a mild acidic pH. From early endosomes, EGF receptors can be either recycled back to the plasma membrane (recycling pathway) or into multivesicular bodies (MVBs) for degradation in lysosomes.^32^ Suppression of SigR1 expression by three different shRNA constructs caused a significant accumulation of EGFR along with p62 and LC3II in A431 cells, confirming that deficiency of SigR1 leads to defective autophagy (Supplementary Figure 1f). Importantly, downregulation of SigR1 did not impair receptor internalization as indicated by similar biotinylation levels of plasma membrane-associated EGFR in SigR1-deficent cells and control cells after 10 min of EGF stimulation (Figure 3a). Next, we monitored the degradation of EGFR after internalization. Interestingly, we observed an efficient degradation of EGFR in control cells after 1 h of activation followed by almost 30% reduction after 3 h, whereas the levels of EGFR in SigR1-deficient cells were unaffected within 1 h after stimulation and only slightly reduced (10–15%) after 3 h. The differences in reduction of EGFR levels after 3 h between control transfected and SigR1-deficient cells were statistically significant (*P*<0.05). Consistent with these data, we found an increased accumulation of LC3II in the SigR1-deficient cells as compared with the controls, again confirming the above-described impairment of lysosomal degradation.

To corroborate these findings, we assessed the fusion of endosomes or autophagosomes to lysosomes using NIH-3T3 cells stably expressing a tandem fluorescence-tagged autophagosomal marker in which LC3 was linked to both monomeric red fluorescent protein (mCherry) and GFP. Because GFP signal is lost upon degradation, this fusion protein serves as a reliable tool for monitoring autophagosome fusion with lysosomes by determining the extent of the colocalization of RFP and GFP signals.^33^ By using live cell imaging (Figure 3c and Supplementary Movie 1), we found that the fusion of autophagosomes to lysosomes was significantly impaired in SigR1-deficient cells as indicated by the minimal loss of GFP fluorescence and maximal colocalization of RFP and GFP signals (Figures 3d and e and Supplementary Movie 1). In most of the control cells, in contrast, we observed a clear reduction of GFP fluorescence and RFP–GFP colocalization. As a final confirmation of the above-described results, we next analyzed the autophagosome to lysosome fusion by EM. We consistently observed the accumulation of several double-membrane autophagosomes (AV) filled with cargos (Supplementary Figures 3a–d) that failed to fuse with lysosomes (Lys) in SigR1-deficient GFP–RFP cell lines (Figure 3f and Supplementary Figures 3b and c) and in SigR1-deficient NSC34 and HEK293 cell lines (Supplementary Figures 2B–D). Taken together, these findings support the notion that SigR1 regulates the endolysosomal pathway by controlling the delivery to and fusion of cargo vesicles with lysosomes.

### Depletion of SigR1 leads to impaired ER to Golgi transport of VSVG-GFP

**Figure 4:**
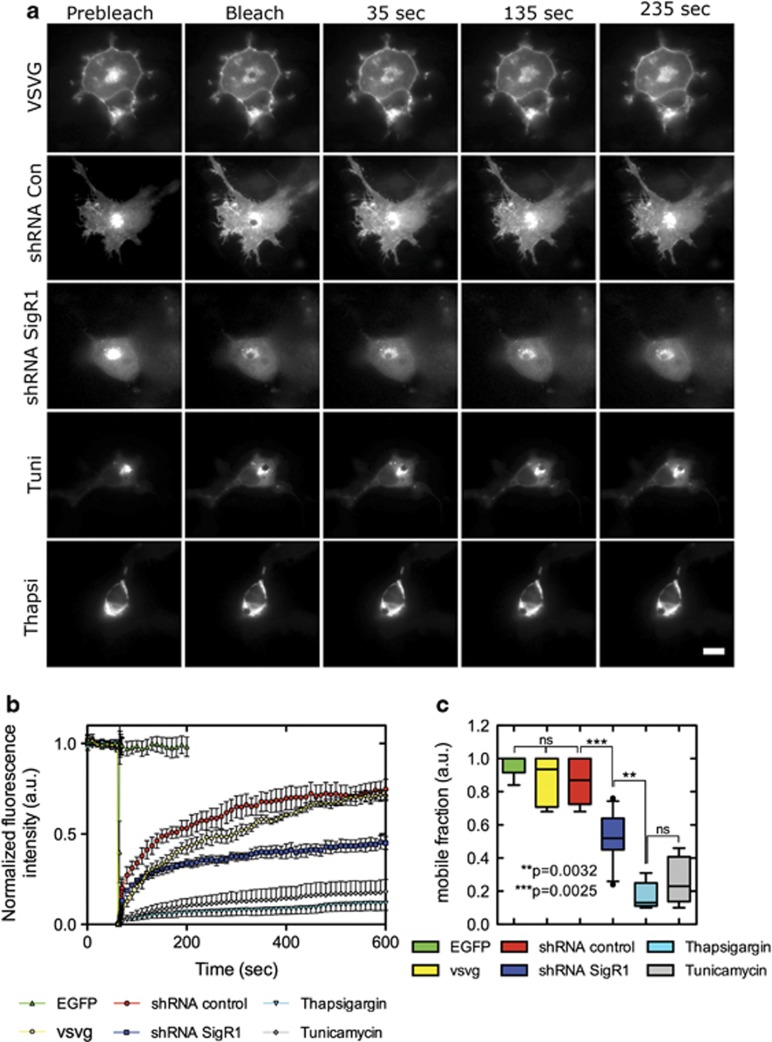
Loss of function of SigR1 leads to impaired ER to Golgi transport of VSVG-GFP. (**a**) Cos7 cells were transfected with VSVG-GFP alone (upper panel) or co-transfected with shRNA control or SigR1 shRNA (as indicated in the figure panels). At 48 h after transfection, the cells were either treated with ER stressor thapsigargin (5 *μ*M) or tunicamycin (10 *μ*M), or left untreated (as indicated in the figure panels). Fluorescence associated with the Golgi complex was photobleached with high-intensity laser light. Subsequent inward delivery of VSVG-GFP from pre-Golgi intermediates was monitored for the indicated periods of time. Scale bar=10 *μ*m. (**b**) Fluorescence recoveries after photobleaching curves of SigR1 shRNA-transfected cells as well as cells treated with thapsigargin and tunicamycin show a clear decrease of the mobile fraction as compared with the shRNA and VSVG controls. Error bars indicate the S.E.M. (**c**) Comparison of the mobile fractions in controls and SigR1-deficient cells. In the box plots, the line in the middle of the box indicates the median; the top line indicates the 75th quartile, whereas the bottom line indicates the 25th quartile. Whiskers represent the 10th (lower) and 90th (upper) percentile, respectively. **P=0.0032; ***P=0.0025

Nascent proteins that leave the ER are channeled through the Golgi complex before they are sorted for transport to their various final destinations. Having observed that ER ultrastructure was compromised considerably in SigR1-deficient cells, we hypothesized that the ER defects might be associated with alterations of vesicle transport from ER to Golgi. To test this hypothesis, we used the temperature-sensitive viral glycoprotein VSVG tagged with green fluorescent protein (VSVG-GFP). VSVG-GFP is misfolded and retained in the ER at 40 °C, whereas it acquires a normal conformation at 32 °C, thus being able to leave the ER and move to the Golgi complex along microtubules by using the microtubule minus-end-directed motor complex of dynein/dynactin (Supplementary Figure 4a).^34, 35^ We analyzed the delivery of VSVG-GFP from pre-Golgi intermediates to the Golgi complex by fluorescence recovery after photobleaching (FRAP) imaging in control cells and SigR1-deficient cells. In VSVG controls (COS-7 cells transfected with VSVG-GFP only) and shRNA control cells, the trafficking of VSVG-GFP vesicles was efficient as indicated by the fast recovery of VSVG-GFP after photobleaching (Figures 4a–c and Supplementary Movies 2 and 3). In contrast, in SigR1-deficient cells and cells treated with the known ER stressors Thapsigargin and Tunicamycin, the intracellular movement of VSVG-GFP vesicles was severely impaired, causing a decrease in VSVG-GFP fluorescence recovery (Figures 4a–c and Supplementary Movie 2). Thus, in agreement with our previous work showing ultrastructural abnormalities of the ER/Golgi complex in SigR1-deficient cells,^20^ these observations indicate that SigR1 is essential for the transport of vesicles from the ER to the Golgi complex. Moreover, these results are also consistent with previous studies showing that VSVG transport is impaired by ER stress.^34, 35^

### shRNA knockdown of SigR1 protein destabilizes membrane lipid rafts and associated calcium signaling

**Figure 5:**
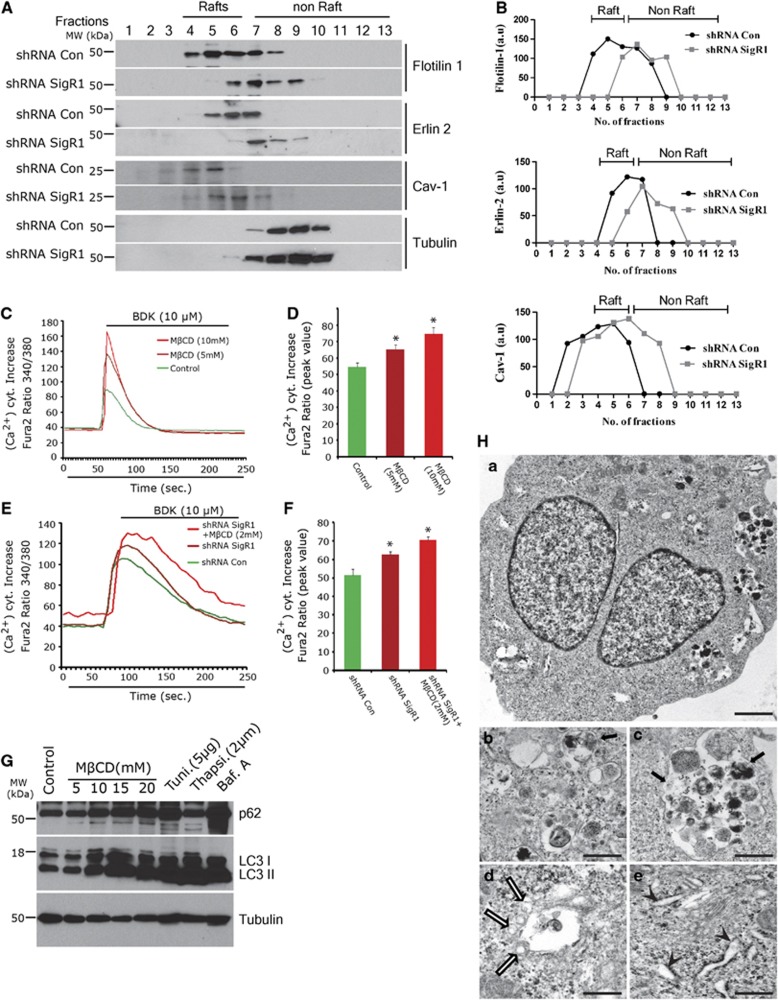
Knockdown of SigR1 protein destabilizes membrane lipid rafts and raft-associated Ca^2+^ signaling. (**A**) NSC34 cells were transiently transfected either with control shRNA or SigR1shRNA as described above. At 48 h after transfection, cell lysates were subjected to sucrose density gradient centrifugation to isolate lipid rafts. Proteins from equal volumes of representative fractions (see also Supplementary Figures 2A and B) were resolved by SDS-PAGE and analyzed by western blotting using specific antibodies against raft markers. (**B**) Quantification of western blots of (**A**). (**C** and **D**) NSC34 cells were treated with M*β*CD for 24 h at the doses indicated. After the treatment, cells were loaded with Fura-2AM for 30 min, washed twice and were then stimulated with 10 *μ*M BDK. Average traces of the BDK-induced increase in [Ca^2+^]^i^ in the transient intracellular Ca^2+^ signaling evoked from the ER store through IP3R. (**E** and **F**) NSC34 cells were transiently transfected with SigR1 shRNA for 48 h and then treated with M*β*CD for 24 h. After the treatment, transient intracellular Ca^2+^ signaling evoked from the ER store through IP3R was measured as described above. Note the increase in basal levels of intracellular calcium in shRNA knockdown cells treated with M*β*CD. Average traces of the BDK-induced increase in [Ca^2+^]^i^ in the knockdown cells and M*β*CD-treated cells as compared with control cells are shown in the pictograph. Results are expressed as mean±S.E.M. of ∼30 cells. Right panels in (**E**) and (**F**) indicate mean changes in peak [Ca^2+^]^i^ measured. The asterisks denote a statistically significant difference (*P<0.05). (**G**) Western blot analysis of NSC34 cells either treated with different doses of M*β*CD or with the ER stressors tunicamycin and thapsigargin or the autophagy inhibitor bafilomycin A. (**H**) NSC34 cells were treated with 5 mM M*β*CD for 24 h, fixed with 2.5% buffered glutaraldehyde and processed for EM. (a) M*β*CD-treated cell showing prominent autophagic vacuoles and MVBs. Scale bar=2.5 *μ*m. (b–e) Higher magnification of the autophagic vacuoles (arrows) of vesicles before fusing with multivesicular body (white arrows) and of the widened ER (arrowheads)

Lipid rafts are microdomains enriched in cholesterol and sphingolipids that – among other functions – orchestrate Ca^2+^ channels to maintain Ca^2+^ mobilization from intracellular stores via the IP3R as well as Ca^2+^ influx across membranes.^36, 37^ SigR1 has been shown to play an important role in modeling lipid rafts and regulating IP3R-mediated Ca^2+^ mobilization at the MAM.^38^ We investigated whether the loss of SigR1 function can affect these processes. Downregulation of SigR1 was found to disrupt lipid rafts by shifting the raft components toward the nonraft fractions. In particular, in SigR1-deficient cells, the raft markers Flotillin-1, Erlin-2 and Caveolin-1 became more enriched in the nonraft fractions (Figures 5A and B). To confirm these findings, we disrupted lipid rafts using mild concentrations of methyl-*β*-cyclodextrin (M*β*CD) that removes cholesterol from lipid rafts.^39^ Treatment with M*β*CD shifted the raft marker vesicle-associated membrane protein-associated protein B (VAPB) to nonraft fractions to a similar extent as in SigR1-deficient cells (Supplementary Figure 5c). Interestingly, treatment with bafilomycin A, which impairs the fusion of autophagosomes to lysosomes, caused alterations similar to the ones we observed after M*β*CD treatment or SigR1 depletion (Supplementary Figure 5c). Moreover, we observed that SigR1 (itself a raft marker) was shifted to and accumulated in nonraft fractions after M*β*CD or bafilomycin A treatment (Supplementary Figure 5c).

Given the crucial role of lipid rafts in maintaining intracellular calcium homeostasis, we examined the consequences of lipid raft disruption on calcium mobilization. We observed a dose-dependent increase of Ca^2+^ release from the ER mediated by the IP3R in cells treated with M*β*CD (Figures 5C and D) that was further increased in amplitude and duration in SigR1-deficient cells (Figures 5E and F).^20^ Given the similar defects in Ca^2+^ signaling and lipid raft structure caused by SigR1 deficiency and M*β*CD treatment and the altered autophagic function in SigR1-deficient cells, we speculated that lipid raft alterations may be associated with alterations of the endolysosomal pathway. We therefore analyzed the levels of autophagy markers in cells pretreated with M*β*CD. Treatment with M*β*CD led to a robust accumulation of the autophagic substrates p62 and LC3II comparable to the accumulation induced by the autophagy inhibitor bafilomycin A and the ER stressors tunicamycin and thapsigargin (Figure 5G) and to that induced by SigR1 deficiency (Figures 2c, d, f and g). Finally, we corroborated these observations by EM analysis. M*β*CD caused the accumulation of various double-membrane autophagosomes filled with autophagic substrates (Figure 5Hb–d), MVBs (Figure 5Hb and c) and of endosomes in the process of merging with autophagosomes (Figure 5Hd, white arrows) as well as widening of the ER (Figure 5He, arrowheads). Similar ultrastructural alterations were also found in SigR1-deficient cells (Figure 1).

Altogether, our findings indicate that SigR1 plays a crucial role in the regulation of the biochemical link between lipid rafts, calcium mobilization, endolysosomal pathways and autophagic processes.

## Discussion

**Figure 6:**
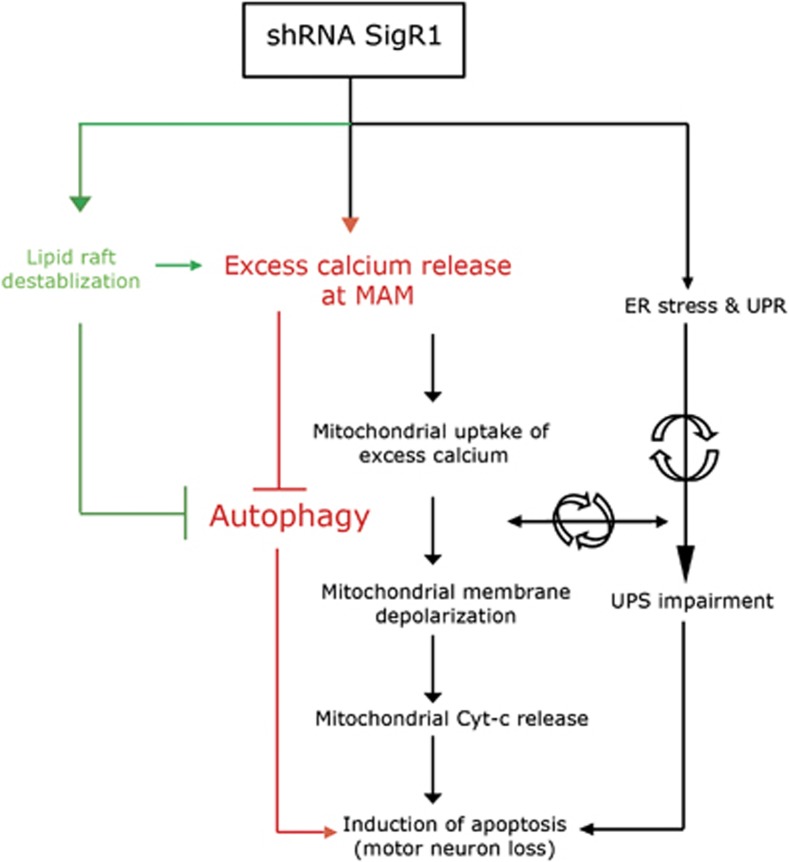
Schematic representation of pathways of autophagy inhibition and apoptosis induction mediated by SigR1 knockdown. Molecular mechanisms corresponding to black lines and arrows were previously described.^20^ As described in the present paper, depletion of SigR1 leads to excess calcium release that induces autophagy inhibition and induction of apoptosis. Alternatively, knockdown of SigR1 leads to the destabilization of lipid rafts that in turn also induces excess calcium release and further autophagy inhibition leading to apoptosis. Lipid raft destabilization can also directly impair the autophagy that can further lead to induction of apoptosis

Recent studies have demonstrated that the SigR1 mutation (E102Q) causes ALS/FTLD in patients and reduces cell viability in cell culture model.^19^ Similarly, a truncated form of the SigR1 (a splice variant generated by splice site at residue E102 of full-length SigR1 protein) promotes mitochondrial energy depletion, ER stress and apoptosis in cell culture models.^40^ Furthermore, aberrant subcellular distribution of altered SigR1 protein was found in motoneurons of sporadic and familial ALS patients.^20^ However, whether mutant SigR1 exerts its pathological effects by a toxic gain- or a loss-of-function mechanism is still an open question. Importantly, SigR1 knockout mice display an ALS-like phenotype by showing motor deficits^22^ and ALS progression exacerbates in SOD1 transgenic mice lacking SigR1.^21, 22^ These findings strongly support loss of function of SigR1 as a causative mechanism of ALS pathogenesis. Consistent with the above-mentioned studies (loss-of-function hypothesis), we previously reported ER stress, mitochondrial abnormalities and apoptosis upon SigR1 depletion in various cell culture models.^20^ In the present study, we provide a more precise molecular mechanism of pathogenesis associated with loss of SigR1 function and propose that depletion of SigR1 involves a vicious circle composed of ER deformation, lipid raft disruption and deranged calcium homeostasis and a defective endolysosomal pathway (see Figure 6).

### SigR1, autophagy and neurodegeneration

Autophagy is considered to be a selective and efficient mechanism for the degradation of misfolded and mutant proteins associated with neurodegenerative diseases including amyloid precursor protein (APP), *α*-synuclein, superoxide dismutase-1 (SOD1) and many others.^41, 42, 43^ Recent reports showed that defects in autophagic flux or in specific autophagy regulatory processes contribute to neurodegeneration and that autophagy is an essential protective mechanism in neurodegenerative disease.^44, 45^ Defective autophagy results in increased autophagosome content and inefficient cargo clearance, leading to the accumulation of damaged organelles and proteins. Moreover, disease proteins such as huntingtin, parkin, Pink-1 and *α*-synuclein alter autophagy by pathological interaction with autophagy regulators.^41, 42, 43, 46^

Consistent with the above studies, we showed by EM that depletion of SigR1 leads to accumulation of autophagosomes (Figure 1 and Supplementary Figures 2 and 3). Autophagy inhibition was confirmed biochemically by western blotting and immunostaining (Figure 2) and also by functional assays that demonstrated autophagosome accumulation, failed fusion with lysosomes and inhibition of EGF–EGFR complex degradation (Figure 3). These results are in line with previous studies showing similar accumulations of autophagosomes and LC3II in cells expressing a truncated form of SigR1 that led to ER stress, mitochondrial abnormalities and apoptosis.^40^ Recent studies in AD models suggest that presenilin-1 (PS1) expression may directly control the acidification of lysosomes through targeting the v-ATPase to these vesicle compartments, thus leading to defective lysosomal proteolysis.^47^ However, we did not observe any significant defects in lysosomal acidification in our preliminary staining experiments using Lysosensor yellow/blue DND-160 (data not shown).

### SigR1 and defective endosomal/vesicle trafficking

Interestingly, knockdown of SigR1 also impairs vesicle trafficking from ER to Golgi as shown by VSVG transport analysis (Figures 4a–c). These results are reminiscent to the phenotypes seen after depletion of cellular proteins involved in endosomal sorting and trafficking, such as Hrs, TSG101 and Vps24.^48, 49^ Endosomal abnormalities are among the earliest pathological features of AD and Down syndrome.^50^ Mutations of Rab7,^51^ of the guanine nucleotide exchange factor alsin^52^ and of Vps54^53^ cause neuropathy and motor neuron diseases in mammals. In addition, mutations in the ESCRT-III complex subunit CHMP2B cause frontotemporal dementia^54^ and dysfunction of the ESCRT-III complex leads to autophagosome accumulation and neurodegeneration.^55^ Taken together, these results provide a link between the dysfunction of the endosomal sorting machinery and neurodegeneration and are consistent with our proposed role of SigR1 as a regulator of endosomal sorting, MVB formation and endosome-to-lysosome trafficking of membrane cargo.

### Lipid raft alterations due to loss of SigR1

Lipid raft domains provide the spatial environment for direct protein–protein interactions and intramolecular cross-talk and function as a unique signal transduction platform.^56^ Lipid raft microdomains regulate several Ca^2+^ channels. Disruption of lipid raft domains inhibits the movement of the calcium sensor stromal interaction molecule 1 (STIM1) into lipid rafts and its association with TRPC1, thereby decreasing TRPC1-dependent store-operated Ca^2+^ entry (SOCE).^57^ We consistently observed that shRNA knockdown of SigR1 disrupts lipid rafts and leads to Ca^2+^ dysregulation as did treatment with M*β*CD (Figure 5). Moreover, in cells treated with M*β*CD, SigR1 protein is shifted to nonraft fractions (Supplementary Figure 5). Lipid rafts stabilize the association of GPI proteins with the ER membrane and are involved in other mechanisms essential for receptor-mediated signal transduction pathways and membrane trafficking.^58, 59^ The defect in ER to Golgi trafficking of VSVG in SigR1 KO cells can also be explained by the fact that SigR1 in lipid rafts plays an important role in vesicular trafficking. This is supported by a study showing similar defects after disruption of lipid rafts.^60^ SigR1 depletion, like M*β*CD treatment, leads to mislocalization of raft components and was accompanied by abnormal accumulation of internal vesicles in MVBs (Figures 5A and G).

### Calcium and neurodegeneration

ER and mitochondria interact physically at the MAM. Ca^2+^ is transferred from the ER to mitochondria in order to stimulate oxidative metabolism; conversely, the metabolically energized mitochondria regulate ER Ca^2+^ homeostasis via the MAM.^18^ SigR1 regulates IP3-mediated Ca^2+^ homeostasis at the MAM. Neuronal intracellular Ca^2+^ signaling is abnormal in many neurodegenerative disorders such as AD, PD, ALS and HD.^61^ Given that neurons are very sensitive to alterations in Ca^2+^ levels, subtle abnormalities in Ca^2+^ signaling may cause neuronal damage in the long term. We previously reported that loss of function of SigR1 increases IP3-mediated ER calcium release and defective ER calcium storage.^20^ In the present study, we provide evidence for a defective ER morphology and altered lipid raft composition after the loss of SigR1 function. We also observed increased Ca^2+^ levels that were further increased by treatment with M*β*CD. These data confirm previous reports that lipid raft microdomains regulate Ca^2+^ mobilization^57^ and suggest that SigR1 is an important constituent of lipid rafts and a regulator of ER Ca^2+^ homeostasis.

## Materials and Methods

### Reagents

Fluorescent nucleic acid stain Hoechst 33258 was purchased from Molecular Probes (Life Technologies GmbH, Munich, Germany). Thapsigargin, tunicamycin, EGF, rapamycin, bafilomycin A1, Fura 2AM, NaCl, KCl, pluronic acid, CaCl2, MgCl2, Glucose, HEPES 4-(2-hydroxyethyl)-1-piperazineethanesulfonic acid, M*β*CD and Bradykinin were purchased from Sigma-Aldrich (St. Louis, MO, USA).

### Antibodies

The antibodies used in this study and their dilutions are described in Supplementary Table 1.

### Cell culture, transient transfection and treatments

#### Cell culture and treatment

Human epithelial cancer cells (HeLa) and motor neuron-like cells (NSC34) were maintained in Dulbecco's modified Eagle's medium (DMEM, Invitrogen, Carlsbad, CA, USA), supplemented with 10% FBS and 1% antibiotic/anti-mycotic solution (Invitrogen). Cell cultures were maintained in a humidified incubator at 37 °C/5% CO_2_. To differentiate NSC34 cells, cells were plated onto poly-D-lysine-coated tissue culture plastic and grown in differentiation medium containing 1 : 1 DMEM/Ham's F12 (Invitrogen), supplemented with 1% FBS, 1% penicillin/streptomycin and 1% modified Eagle's medium with non-essential amino acids (Invitrogen) for 3 days. For the stable cell lines, all cells were grown in a humidified atmosphere at 37  °C with 5% CO_2_. NIH-3T3 cells as well as tandem mCherry/GFP NIH-3T3 cells were cultured in DMEM supplemented with 10% heat-inactivated FCS. Plat-E cells were grown in DMEM supplemented with 10% heat-inactivated FCS, 1% penicillin/streptomycin (Gibco, Life Technologies GmbH), 1 *μ*g/ml puromycin (Sigma-Aldrich) and 10 *μ*g/ml blasticidin (Invitrogen).

#### Generation of stable cell lines with retroviral infection

NIH-3T3 cells stably expressing GFP-LC3 and mCherry-EGFP-LC3B were generated by retroviral infection. Retroviruses were generated by transient transfection of the retroviral producer cell line Plat-E with 20 *μ*g pBABEpuro GFP-LC3,^62^ and pBABEpuro mCherry-EGFP-LC3B,^63^ respectively. After two rounds of infection, retrovirus-expressing NIH-3T3 cells were selected with 3 *μ*g/ml puromycin to generate stable cell pools. For the transfections the cells were seeded without antibiotics.

#### Retroviral infection

The day before transfection, Plat-E cells^64^ were seeded at 2 × 10^6^ cells per 100 mm dish. The next day, pBABE-based retroviral vectors were introduced into Plat-E cells using the Ca^2+^ phosphate precipitation technique. At 6 h after transfection, the medium was replaced and cells were incubated overnight at 37 °C with 5% CO_2_. On the same day, NIH-3T3 were seeded at 8 × 10^5^ cells per 100 mm dish. After 24 h, virus-containing supernatants derived from the Plat-E cultures were filtered through a 0.45 *μ*m PVDF filter (Millipore, Merck Chemicals GmbH, Darmstadt, Germany) and supplemented with 4 *μ*g/ml polybrene (Sigma-Aldrich). NIH-3T3 cells were incubated in the virus/polybrene-containing supernatants overnight. After infection, the cells were re-plated in 10 ml fresh medium. A second round of infection was performed after 24 h to maximize the rate of infection. At 48 h after the last infection, puromycin (3 *μ*g/ml, Sigma-Aldrich) was added to select for stable clones for at least 2 weeks.

### Plasmids and RNA interference

The HuSH shRNA (pRFP-C-RS) retroviral RFP vector containing four unique 29-mer shRNA SigR1 oligoneucleotides (four different shRNA SigR1 constructs) for optimal suppression of SigR1 was purchased from Origene (Rockville, MD, USA). Non-effective 29-mer scrambled shRNA cassette in the same vector was used as a shRNA control in all the experiments. For some experiments, shRNA SigR1 vector was modified to generate a stop codon in the RFP sequence to generate shRNA SigR1 without the RFP tag. Knockdown by transient transfections of shRNA SigR1 in all the cell lines used was performed using the Lipofectamine 2000 reagent (Invitrogen) according to the manufacturer's recommendations. Western blots were performed to verify the efficiency of the knockdown (see below). VSVG-GFP was a kind gift from Professor Jennifer Lippincott-Schwartz.^34, 35^

### Immunocytochemistry

HeLa cells or NIH-3T3 cells were cultured on *μ*-dish (ibidi, GmbH, Am Klopferspitz, Germany) and transiently transfected either with shRNA SigR1 alone or together with GFP-LC3 constructs. The medium was changed after 4 h. After 48 h, cells were fixed in 4% PFA and processed for confocal microscopy. In further experiments after 48 h of knockdown, cells were permeabilized with 0.1% Triton X-100, fixed in 4% PFA and immunostained (see Supplementary Table 1 for antibody dilutions). Secondary Alexa488- or Alexa594-conjugated anti-mouse or anti-rabbit antibodies (Invitrogen) were used for visualization. Nuclei were stained with Hoechst 33342 (1 *μ*g/ml). Samples were mounted with fluorescent mounting media (DAKO, Glostrup, Denmark) and visualized using a Zeiss LSM 700 confocal microscope (Zeiss, Göttingen, Germany). Resulting images were processed using the Zeiss LSM software and Adobe Photoshop CS5 (Adobe Systems GmbH, Munich, Germany).

### Fura-2 calcium imaging

The intracellular calcium ion concentration, [Ca^2+^]^i^, was measured using a conventional Fura-2 technique.^65^ After 48 h of SigR1 knockdown, NSC34 cells in glass bottom *μ*-dishes (ibidi, GmbH) were loaded with the membrane-permeable AM form of Fura-2 (1.5 ng/*μ*l; Invitrogen) in the presence of pluronic acid (25%) for 30 min at 37 °C. Emitted fluorescence at 530 nm (detected using a Sensicam; pco.imaging, PCO AG, Kelheim, Germany) in response to alternate excitation at 340 and 380 nm (using the Polychrome V monochromator; TILL Photonics, FEI Munich GmbH, Germany) was used to measure intracellular Ca^2+^ concentrations. Data were expressed as emission ratios in response to 340 nm/380 nm excitation. Whole-cell calcium measurements from SigR1-deficient and control NSC34 cells were obtained at room temperature (23–25 °C). During imaging, cells were kept in a bathing solution containing 100 mM NaCl, 5.4 mM KCl, 2 mM CaCl_2_, 1 mM MgCl_2_, 10 mM HEPES, 10 MES, 5.5 mM glucose and pH was adjusted to 7.4.

### Transmission electron microscopy

Hek293 cells were transfected with control, scrambled shRNA and SigR1 shRNA as described above. Cells were collected by scraping, washed in 0.1 M phosphate buffer and immediately fixed with 2.5% glutaraldehyde in 0.1 M phosphate buffer for 24 h followed by washing in buffer for further 24 h. Cell pellets were collected by centrifugation (1000 r.p.m., 5 min) and embedded in 2% agarose (at 60 °C; Fluka 05073, St. Louis, MO, USA). Small blocks of embedded cells were sliced and post-fixed in 2.5% glutaraldehyde for 24 h followed by washing in 0.1 M phosphate buffer for 24 h. Agarose blocks were then incubated in 1% OsO_4_ (in 0.2 M phosphate buffer) for 3 h, washed twice in distilled water and dehydrated using ascending alcohol concentrations (i.e., 25, 35, 50, 70, 85, 95 and 100% each step for 5 min). Dehydrated blocks were incubated in propylenoxide followed by subsequent 20 min of incubation in a 1 : 1 mixture of epon (47.5% glycidether, 26.5% dodenylsuccinic acid anhydride, 24.5% methylnadic anhydride and 1.5% Tris (dimethylaminomethyl) phenol) and propylenoxide. The samples were then incubated in epoxy resin for 1 h at room temperature followed by polymerization (28 °C for 8 h, 80 °C for 2.5 h and finally at room temperature for 4 h). Ultra-thin sections (70 nm) were mounted on grids, contrast enhanced with uranyl acetate and lead citrate and examined with a Philips EM 400 T electron microscope (Philips GmbH, Germany) as previously described.^66^ The images were captured using a Morada digital camera (Olympus, Japan).

### Fluorescence recovery after photobleaching

For live cell imaging experiments, Cos7 cells were seeded onto homemade glass-bottomed dishes (6 cm), and co-transfected either with shRNA SigR1/VSVGts045-GFP or shRNA control VSVGts045-GFP. VSVGts045-GFP was allowed to accumulate in the ER at 42 °C for 8 h and cells were imaged on an Axio Observer Z1 inverted microscope equipped with heating stage and CO_2_ controller (Zeiss) maintained at a constant temperature of 32 °C. A portion of the ER (approximately ø 3.84 *μ*m) in the periphery of the cell was photobleached using a 405 nm laser driven by the UGA-40 control unit (Rapp Opto Electronic GmbH, Wedel, Germany). The recovery of the fluorescent signal was monitored by imaging the cells every second for 15 min. Imaging was done using an Evolve EM-CCD camera driven by ZEN software (Zeiss). For all experiments the area bleached and the duration and intensity of the laser impulse was kept constant. The extent of recovery of the fluorescent signal was determined using ImageJ (developed by Rasband, W.S., National Institutes of Health, Bethesda, MD, USA, http://imagej.nih.gov/ij/) to measure the average pixel intensity values within three distinct regions of interest (ROIs): ROI1: bleached area; ROI2: unbleached area within the cell; and ROI3: background. Normalized FRAP recovery curves and the mobile fractions were calculated using the program easy FRAP.^67^

### Live cell imaging to analyze RFP–GFP–LC3 fusion

To analyze the dynamics of the RFP–GFP–LC3 fusion protein, GFP and RFP channels were acquired every min for up to 4 h using the imaging system described above. The extent of autophagosome maturation was determined by measuring the colocalization of the GFP and RFP signals as expressed by Pearson's coefficient using ZEN software.

### Western blot analysis

Cells were washed with ice-cold PBS twice, scraped off the culture plate and resuspended in lysis buffer (0.5% Triton X-100 in PBS, 0.5 mM PMSF and complete protease inhibitor mixture, Roche Applied Sciences, Roche Diagnostics GmbH, Mannheim, Germany). After incubation on ice for 30 min, lysates were briefly sonicated. Clear lysates were obtained after centrifugation for 5 min at 6000 r.p.m. Protein concentrations were determined using the BCA method (Molecular Probes). Equal amounts of protein were boiled for 5 min in 2 × SDS sample buffer and subjected to 10 or 12% SDS-PAGE before transfer to a polyvinylidenedifluoride (PVDF) membrane. The membranes were blocked in 5% skimmed milk in 0.05% Tween 20/Tris-buffered saline (TBS-T) for 30 min before incubation with primary antibody. Primary antibodies were used in a dilution of 1 : 1000. The membranes were incubated overnight at 4 °C, then washed 3 times in TBS-T and incubated for 1 h with appropriate horseradish peroxidase-conjugated secondary antibody (dilution 1 : 10 000, Thermo Scientific, Waltham, MA, USA). Immunoreactive proteins were detected by enhanced chemiluminescence (Amersham Biosciences, GE Healthcare, Life Sciences, Germany). Densitometric quantification of the band intensity was normalized to tubulin levels using Adobe Photoshop CS5.

### EGFR endocytosis and degradation assay

For EGFR endocytosis and degradation analysis, A431 cells were transfected with either control or SigR1 shRNA, and 48 h later, the cells were starved in DMEM with 0.1% BSA for 4 h. After starvation, cells were treated with EGF (100 *μ*g for 10 min) to stimulate EGFR endocytosis. Cells were then collected at various time points (as indicated in Figure 3b) and lysed in RIPA buffer (50 mM Tris-HCl, pH 7.5, 1% Triton X-100, 150 mM NaCl, 1 mM EDTA and 0.1% Na deoxycholate) with protease inhibitor. Protein extracts were resolved by SDS-PAGE and immunoblotted using anti-EGFR antibody.

### EGFR surface biotinylation assay

Surface biotinylation assays were performed as previously described^68^ with minor modifications. Briefly, A431 cells were transfected with either control or SigR1 shRNA, and 48 h later, the cells were starved in DMEM with 0.1% BSA for 4 h. After starvation, cells were treated with EGF (100 *μ*g for 10 min) to stimulate EGFR endocytosis and then proceeded for surface biotinylation assay.

### Membrane lipid raft analysis

Membrane lipid rafts were prepared as described previously^57^ with some modifications. Briefly, NSC34 cells transfected with SigR1 shRNA or control shRNA either treated with M*β*CD or left untreated were washed with phosphate-buffered saline, pH 7.4, and lysed for 30 min on ice in pre-chilled TNE buffer (1% v/v Triton X-100, 25 mMTris-HCl, 150 mM NaCl, and 5 mM EDTA pH 7.5) supplemented with protease and phosphatase inhibitors (Roche Applied Science). Lysates were homogenized using a Dounce homogenizer (VWR International GmbH, Langenfeld, Germany) followed by a brief centrifugation. Equal amount of proteins were quantified by BCA method (Supplementary Figure 2A). Then, 800 *μ*l of the postnuclear supernatant (PNS) was mixed with an equal volume of 80% sucrose (w/v) and overlaid with 2 ml of 35% sucrose followed by 1 ml of 5% sucrose (in TNE buffer). A total of 5 ml of samples was centrifuged at 39 000 r.p.m. for 18 h at 4 °C in a SW 55 Ti rotor (Beckman Coulter GmbH, Krefeld, Germany). Then, 13 equal fractions (320 *μ*l) were collected from the top of the tube and proteins were precipitated using ice-cold acetone. Samples were then mixed with SDS sample buffer and analyzed by western blotting. Coomassie blue staining showed equal loading of either control shRNA or SigR1 shRNA fractions (Supplementary Figures 4b and c).

### Statistical analysis

Except for the FRAP and colocalization analyses, we used the unpaired Student's *t*-test for the comparison of the means of two samples. Samples were considered statistically different if *P*<0.05. In all graph, data are represented as mean±S.D. of three independent experiments. For Ca^2+^ measurements, results are expressed as mean±S.E.M. of ∼30 independent measurements.

For the FRAP and colocalization experiments (Figures 3 and 4), all graphs and statistical analyses were done using Prism 5 (GraphPad Software, Inc., La Jolla, CA, USA). A total of 5 to 6 independent experiments were analyzed. Differences among the various samples were determined using the two-tailed Mann–Whitney nonparametric *U*-test. The null hypothesis (the two groups have the same median values, i.e., they are not different) was rejected when *P*>0.05.

## Abbreviations

ALS: amyotrophic lateral sclerosis
FTLD: frontotemporal lobar degeneration
MVBs: multivesicular bodies
EGFR: epidermal growth factor receptor
FRAP: fluorescence recovery after photobleaching
Lys: lysosomes
M*β*CD: methyl-*β*-cyclodextrin
APP: amyloid precursor protein
SOD1: superoxide dismutase-1
