# Supplementary figures and images for: Loss of function of the ALS protein SigR1 leads to ER pathology associated with defective autophagy and lipid raft disturbances
Source: Cell Death Dis. 2014 Jun 12;5(6):e1290–. doi: 10.1038/cddis.2014.243 (PMC4611717; doi:10.1038/cddis.2014.243)

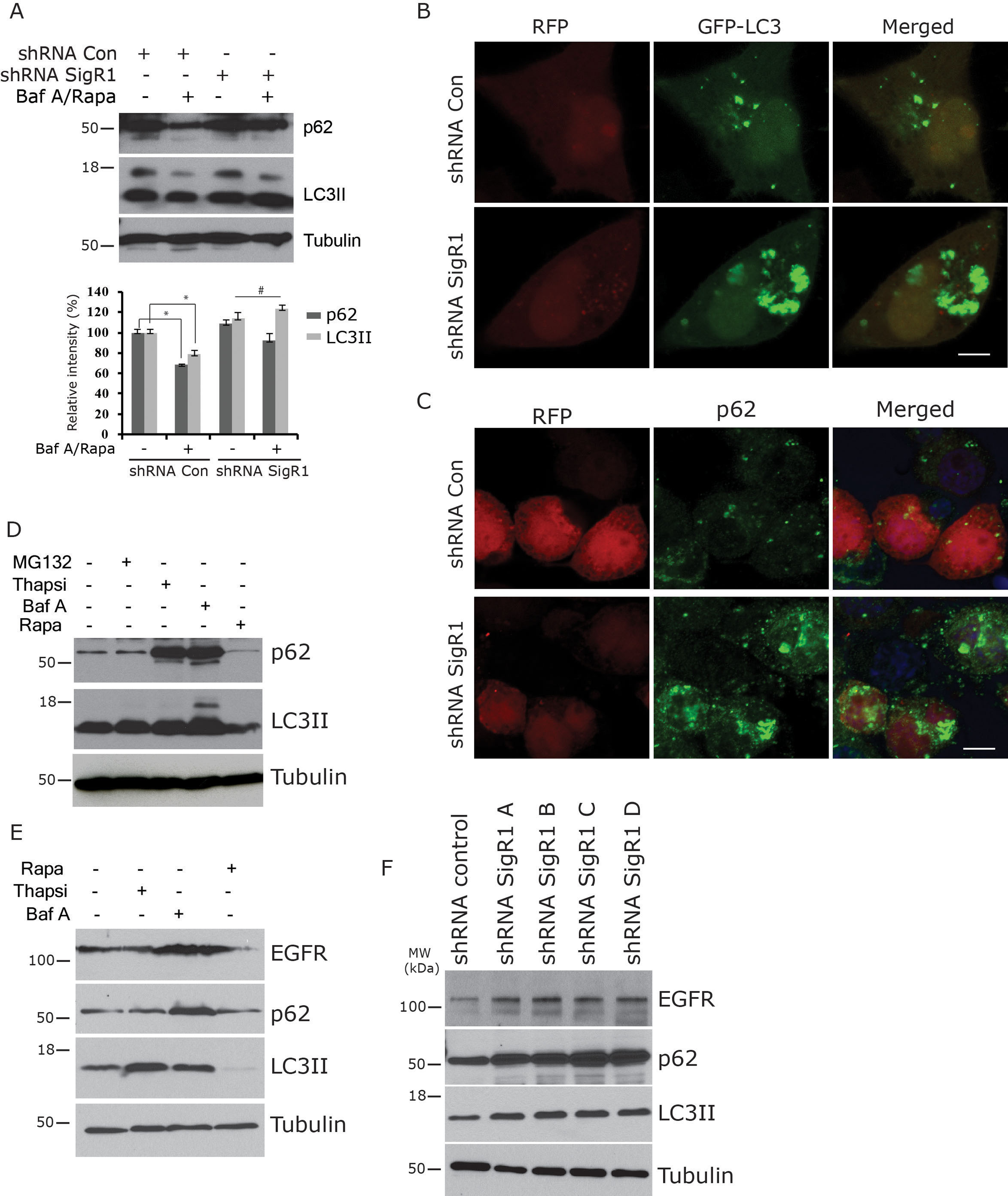

Supplement: Supplementary Figure 1 [file cddis2014243x1.tif]

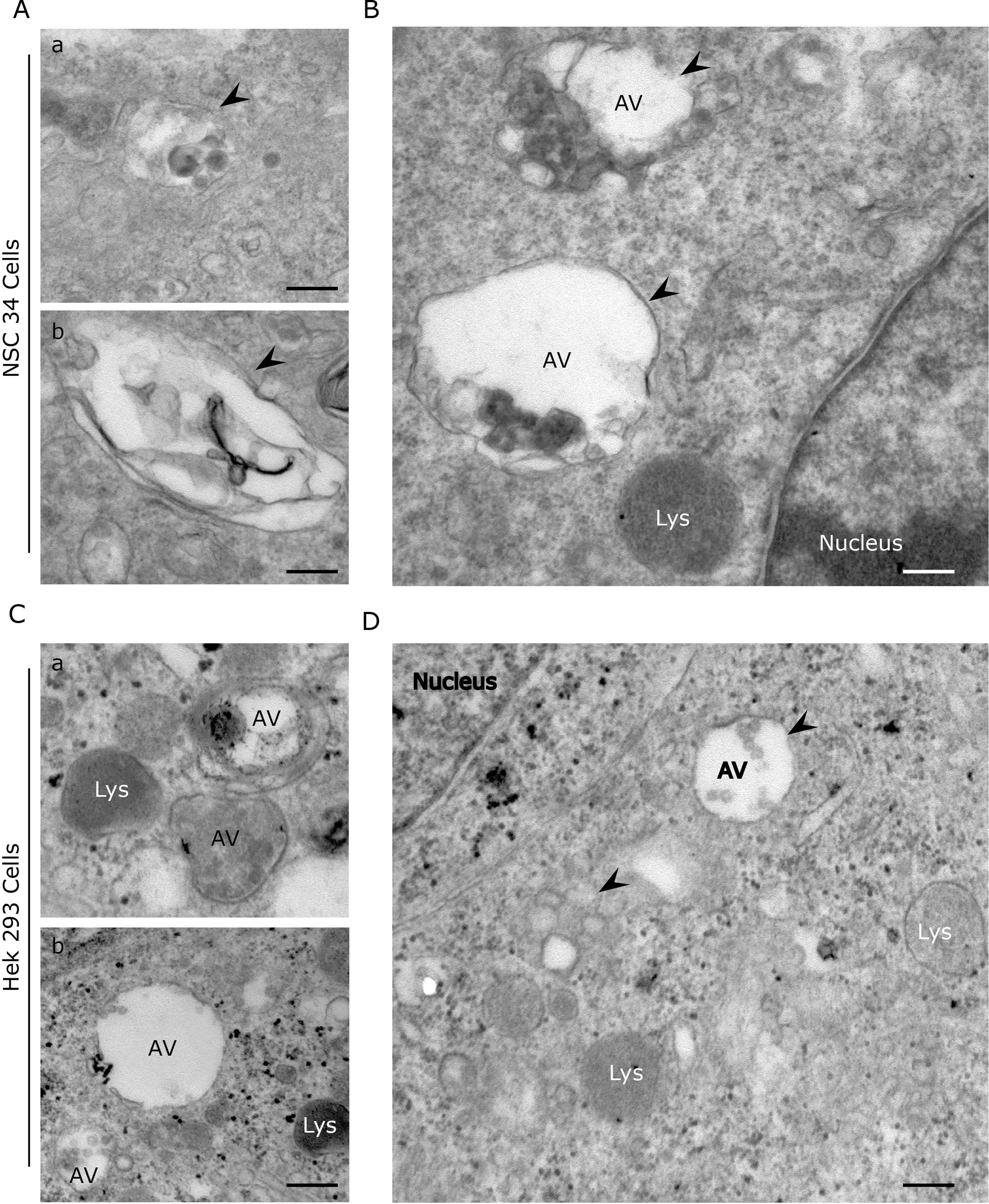

Supplement: Supplementary Figure 2 [file cddis2014243x2.tif]

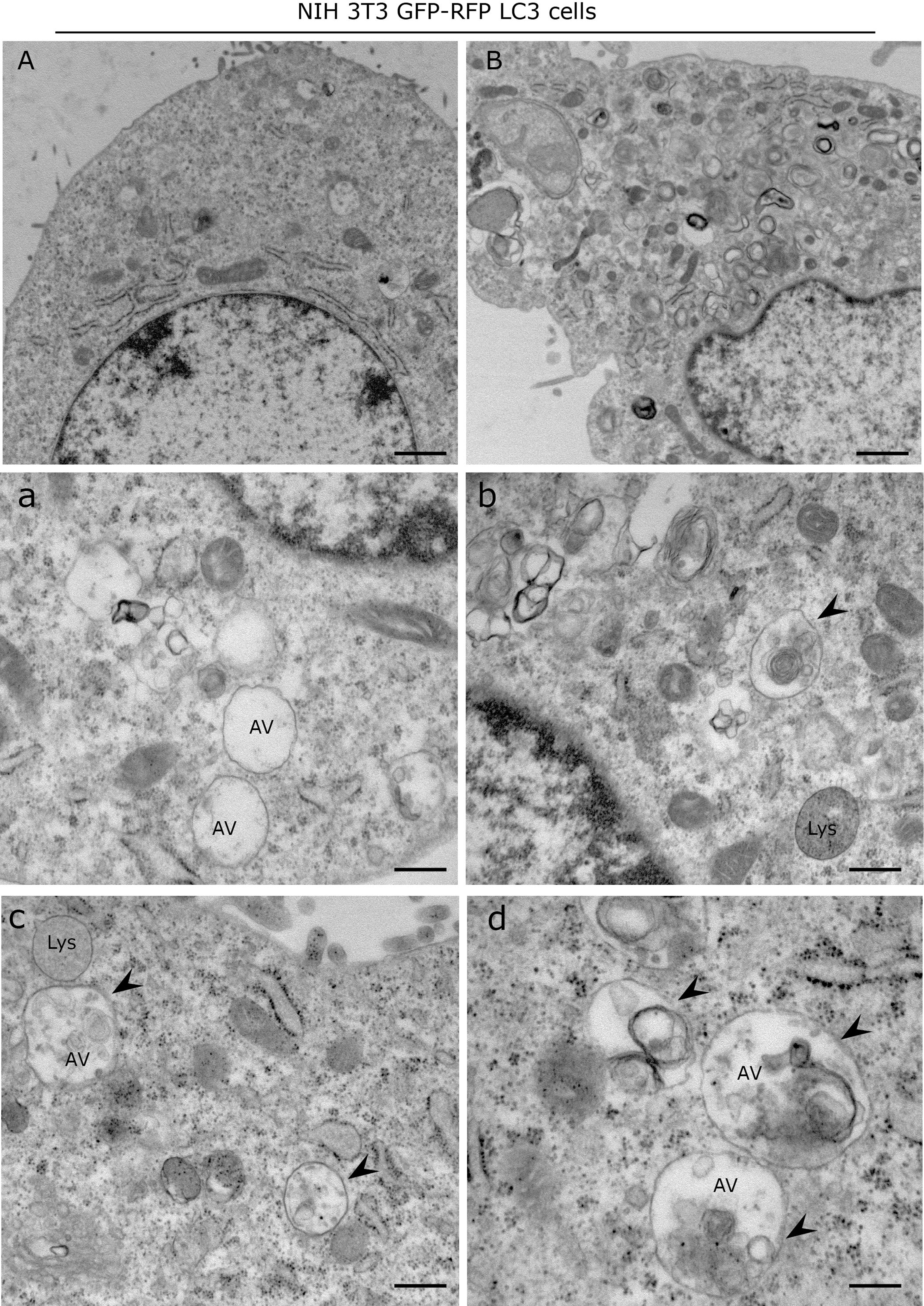

Supplement: Supplementary Figure 3 [file cddis2014243x3.tif]

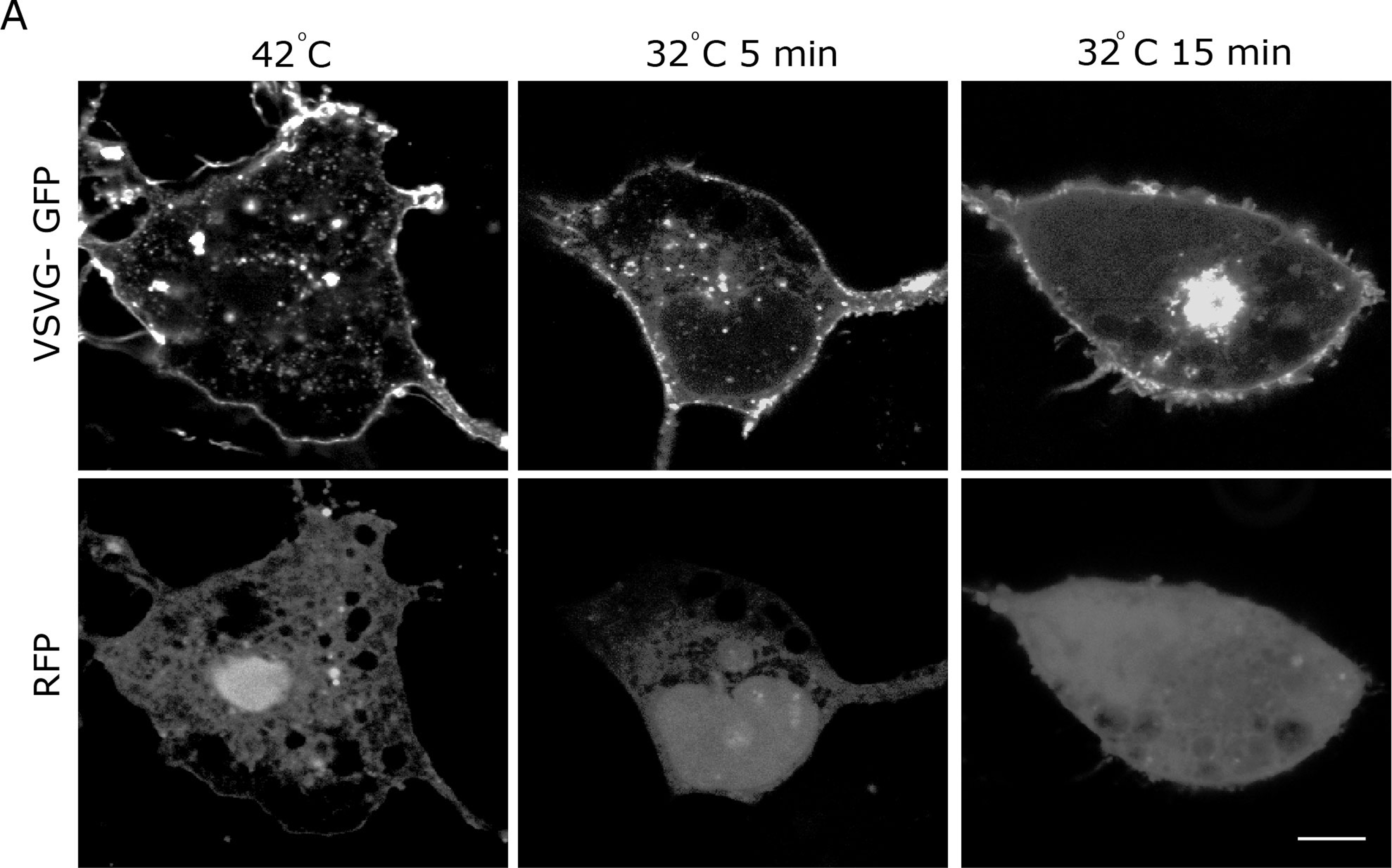

Supplement: Supplementary Figure 4 [file cddis2014243x4.tif]

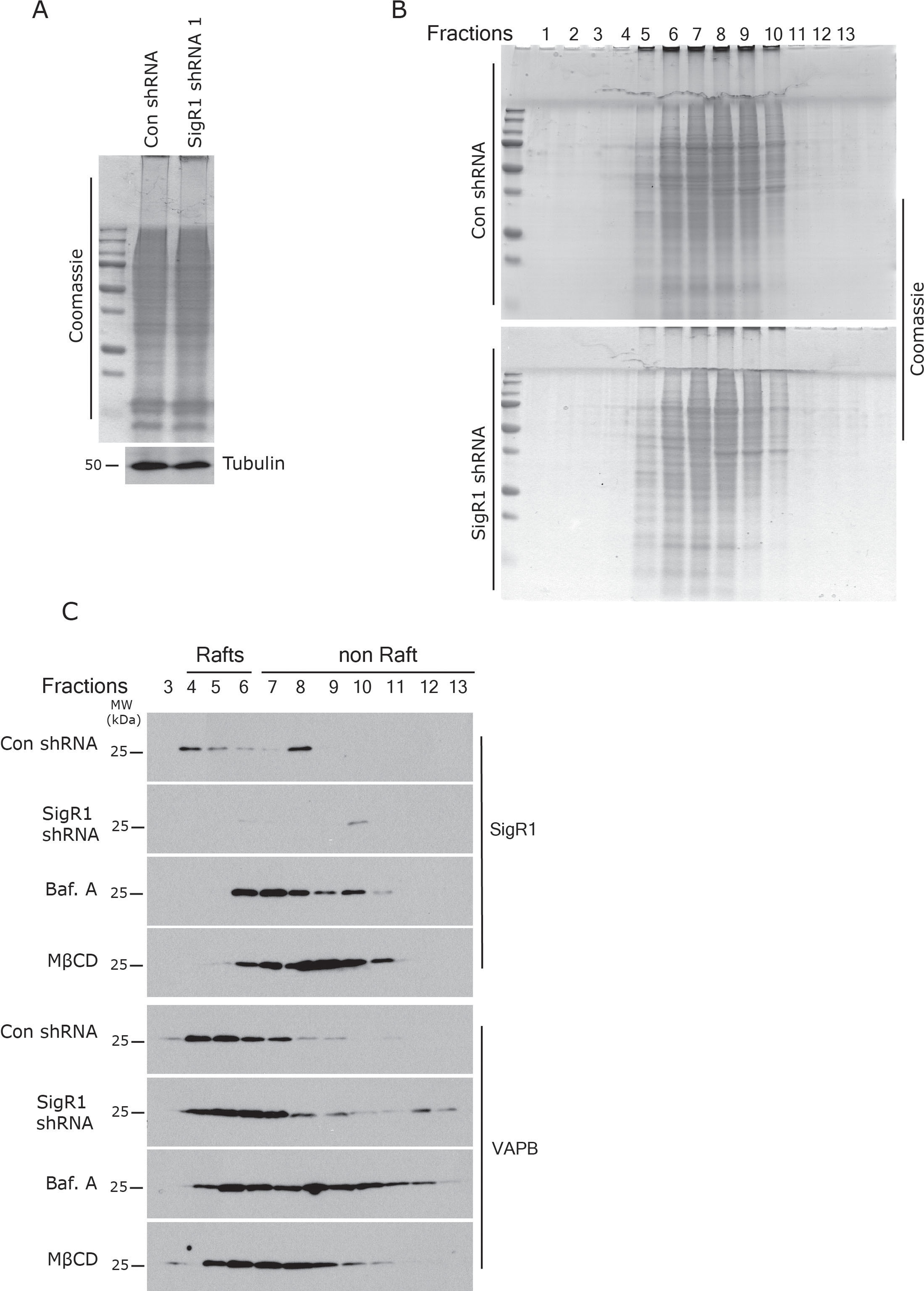

Supplement: Supplementary Figure 5 [file cddis2014243x5.tif]
