# Supplementary material for: Loss of function of the ALS protein SigR1 leads to ER pathology associated with defective autophagy and lipid raft disturbances
Source: Cell Death Dis. 2014 Jun 12;5(6):e1290–. doi: 10.1038/cddis.2014.243 (PMC4611717; doi:10.1038/cddis.2014.243)
Supplement: Supplementary Table 1 [file cddis2014243x6.doc]

**Supplementary table 1**

| **Antibodies** | **Source** | **Dilution ( WB)** | **Species** |
| --- | --- | --- | --- |
| Erlin 2 | Sigma Aldrich | 1:1000 | Rabbit |
| Flotilin 1 | cell Signalling | 1:500 | Mouse |
| Caveolin 1 | cell Signalling | 1:500 | Mouse |
| EGFR | cell Signalling | 1:500 | Mouse |
| LC3 | MBL | 1:2000 | Rabbit |
| p62 | MBL | 1:500 | Rabbit |
| GADD 153 | Santa Cruz | 1:1000 | Mouse |
| Sigma receptors-1 | Santa Cruz | 1:500 | Mouse |
| Rab7 | Sigma Aldrich | 1:1000 | Rabbit |
| Hsp70 | Santa Cruz | 1:1000 | Mouse |
| GRP78 | cell Signalling | 1:500 | Mouse |
| EEA1 | cell Signalling | 1:500 | Mouse |
| α tubulin | Sigma | 1:10000 | Mouse |
| GADD 153 | Santa Cruz | 1:1000 | Rabbit |
| human specific Sigma receptors-1 | sigma | 1:1000 | Rabbit |
|  |  |  |  |
