# Supplementary material for: Loss of function of the ALS protein SigR1 leads to ER pathology associated with defective autophagy and lipid raft disturbances
Source: Cell Death Dis. 2014 Jun 12;5(6):e1290–. doi: 10.1038/cddis.2014.243 (PMC4611717; doi:10.1038/cddis.2014.243)
Supplement: Supplementary Information [file cddis2014243x10.doc]

**Suppl. Figure legends**

**Suppl. Fig. 1: Loss of function of SigR1 impairs autophagy.**

**(A)** NSC34 cells were transiently transfected as described. 48 h later cells were treated with or without the autophagy inhibitor Bafilomycin A (20 nM) for 3 h, which was then replaced by the autophagy inducer rapamycin (20 nM) for 3 additional h. Cell lysates were prepared and subjected to immunoblot analysis using p62 and LC3 antibodies. Quantification: panel below. Values are means ± SD of three independent experiments. * *P <*0.05; ^#^not significant. **(B)** HeLa cells were transiently co-transfected with either control or SigR1 shRNA and GFP-LC3. 48 h after transfection cells were processed for immunofluorescence. Representative images depict the accumulation of GFP-LC3 as a marker for autophagy inhibition. **(C)** HeLa cells were transiently transfected with either control or SigR1 shRNA. 48 h after transfection cells were processed for immunofluorescence. Representative images depict the accumulation of p62 as a marker for autophagy inhibition. **(D)** NIH-3T3 cells were treated with or without the autophagy inducer rapamycin (20 nM), the autophagy inhibitor bafilomycin A (20 nM) and thapsigargin (5 µM) and incubated for 4 h. Cell lysates were prepared and subjected to immunoblot analysis using p62 and LC3 antibody to confirm autophagic flux in these cells. **(E)** A431 cells were treated accordingly. Cell lysates were prepared and subjected to immunoblot analysis using p62, LC3 and EGFR antibodies to confirm autophagic flux alterations in these cells. **(F)** A431 cells were transiently transfected with various SigR1 shRNA constructs. 48 h after transfection cell lysates were subjected to immunoblot analysis using p62, LC3 and EGFR antibodies to confirm autophagy inhibition.

**Suppl. Fig. 2: Depletion of SigR1 leads to the accumulation of autophagic vacuoles which failed to fuse with lysosomes.** HEK293 or NSC34 motor neuronal like cells were transfected either with control shRNA or SigR1 shRNA. 48 h later the transfected cells were fixed with 2.5% buffered glutaraldehyde and processed for EM. **(A)** NSC34 motor neuron-like cell transfected with SigR1 shRNA shows defective autophagy **(a, b)** with accumulation of autophagosomes (black arrowhead). **(b)** Enlarged view of one of the autophagic vacuoles. Scale bar = 0.2 µm. (**B)** Large, double membrane-bound autophagic vacuole (AV) containing membranous and other osmiophilic accumulations (arrowheads). Note the absence of fusion between AV and a nearby lysosome (Lys). Scale bar = 0.25 µm. **(C)** Enlarges view of membrane-bound autophagic vacuole (AV) containing osmiophilic accumulations **(a, b)** which are not fused with an adjacent lysosome (Lys). Scale bar = 0.25 µm. **(D)** Autophagic vacuole (AV) (arrowhead) and other small membranous vacuoles (arrows), probably endosomes not fused with nearby lysosomes (Lys). Scale bar = 0.25 µm.

**Suppl. Fig. 3: Autophagic degradation defects due to SigR1 depletion of in NIH3T3 GFP-RFP cell lines.** NIH3T3 GFP-RFP cell lines were either transfected with control shRNA **(A)** or SigR1 shRNA **(B).** The transfected cells were processed for EM 48 h later. **(a-d)** Enlarges views of cells depleted of SigR1 (**a)** Accumulation of several double membrane-bound autophagosomes. Scale bar = 0.5 µm. **(b)** Several autophagic vacuole (AV) (arrowheads) containing membranous and other osmiophilic accumulations which are not fused with a nearby lysosome (Lys). Scale bar = 0.4 µm. **(c)** Double membrane-bound autophagic vacuoles (AV) containing osmiophilic material. Note the absence of fusion between AV and a nearby lysosome (Lys). Scale bar = 0.4 µm. **(d)** Autophagic vacuoles (AV) containing membranous and other osmiophilic accumulations. Scale bar = 0.3 µm.

**Suppl. Fig. 4:** Cos7 control cells transiently transfected with VSVG–GFP were incubated at 42°C to retain VSVG–GFP in the ER (left panel) and then shifted to 32°C for 5 min (middle panel) or for 15 min (right panel), fixed in 4% formaldehyde and analyzed. Scale bar = 5 µm.

**Suppl. Fig. 5: Loss of function of SigR1 destabilizes lipid rafts. (A)** NSC34 cells were either transiently transfected with control or SigR1shRNA as described above. 48 h after transfection, cell lysates were prepared and equal amounts of protein were quantified by BCA. Coomassie staining depicts the equal amounts of protein prior to density gradient centrifugation to isolate lipid rafts (see methods for details).

**(B)** NSC34 cells were either transiently transfected with control or SigR1 shRNA as described above or treated with MβCD for 24h and with the autophagy inhibitor Bafilomycin A (20 nM) for 4 h. Proteins from equal volumes of representative collected fractions were separated by SDS-PAGE and analyzed by Western blotting using specific antibodies against the indicated raft markers.

**(C)** Coomassie staining showing the equal amounts of protein in control and SigR1 shRNA fractions collected after the density gradient centrifugation.

**Movie legends**

**Movie 1.** SigR1 down regulation impairs autophagosomal degradation. Note the higher degree of co-localization between the RFP and GFP signals in SigR1-deficient cells. Play back speed is approximately 900-fold of the real speed.

**Movie 2.** SigR1 down regulation impairs vesicle transport from ER to Golgi. Note the slower recovery of the fluorescence signal within the bleached area in SigR1-deficient cells and cells treated with thapsigargin and tunicamycin as compared to shRNA control cells. Play back speed is approximately 15-fold of the real speed.

**Movie 3.** Fluorescence recovery after photobleaching in control cells expressing GFP alone or VSVG-GFP. Note the very fast recovery of the fluorescence intensity within the bleached area. Play back speed is approximately 15-fold of the real speed.

**Suppl. table legend**

The table shows the antibodies used in this study and their respective dilutions and sources.
